# Potential risks and solutions for sharing genome summary data from African populations

**DOI:** 10.1186/s12920-019-0604-6

**Published:** 2019-11-04

**Authors:** Nicki Tiffin

**Affiliations:** 1 Computational Biology Division, Integrative Biomedical Sciences, University of Cape Town, Cape Town, South Africa; 2 Wellcome Centre for Infectious Disease Research in Africa, Institute of Infectious Diseases and Molecular Medicine, University of Cape Town, Cape Town, South Africa; 3 Centre for Infectious Disease Epidemiology Research, School of Public Health and Family Medicine, University of Cape Town, Cape Town, South Africa

**Keywords:** African genomes, Genome summary results, African diversity, Community harms

## Abstract

Genome data from African population can substantially assist the global effort to identify aetiological genetic variants, but open access to aggregated genomic data from these populations poses some significant risks of community- and population- level harms. A recent amendment to National Institutes of Health policy, following various engagements with predominantly North American scientists, requires that genomic summary results must be made available openly on the internet without access oversight or controls.

The policy does recognise that some sensitive, identifiable population groups might be harmed by such exposure of their data, and allows for exemption in these cases. African populations have a very wide and complex genomic landscape, and because of this diversity, individual African populations may be uniquely re-identified by their genomic profiles and genome summary data. Given this identifiability, combined with additional vulnerabilities such as poor access to health care, socioeconomic challenges and the risk of ethnic discrimination, it would be prudent for the National Institutes of Health to recognise the potential of their current policy for community harms to Africans; and to exempt all African populations as sensitive or vulnerable populations with regard to the unregulated exposure of their genome summary data online.

Three risk-mitigating mechanisms for sharing genome summary results from African populations to inform global genomic health research are proposed here; namely use of the Beacon Protocol developed by the Global Alliance for Genomics and Health, user access control through the planned African Genome Variation Database, and regional aggregation of population data to protect individual African populations from re-identification and associated harms.

## Background

Because of the complexity and depth of African genomes compared to rest-of-world populations, genome summary data that include population allele frequencies from African populations can greatly enhance identification of disease-causing and other variants in African as well as rest-of-world research, and advances in health genomics research on the African Continent can contribute meaningfully to biomedical research globally [1].

Since 2008, genomic summary results (GSR) had been archived in controlled-access portions of NIH-designated data repositories due to concerns that an individual’s inclusion in a group could be ascertained given their whole genome data [2]. In November 2018, the National Institutes of Health (NIH) released a statement updating their policy on management of access to GSR, based on recent workshops and various engagement mechanisms undertaken in the USA to explore access options for sharing GSR.

The NIH concluded that respondents in general believed that benefits of open access to GSR outweigh the risks. This informed the subsequent NIH requirement that GSR generated with NIH funding should be made freely available on the internet with no access restriction – with the caveat that some sensitive population groups could be exempt from this requirement due to a risk of stigmatisation of specific communities or populations. This amended policy also applies to research programs in Africa that are funded by the NIH, and it is important to review how the policy might affect the protection of African study participants and their communities, particularly as it appears that there was no documented engagement with African stakeholders when considering the amendment of the policy.

According to documented elements of the public engagement process, the NHGRI Workshop on Aggregate Genomic Data (May 2016), had predominantly North American attendees and no registered African representatives [3]. A “Request for Information” call in 2017 [4] recorded responses from 109 parties (37 of whom appeared to be users of ExAC and gnomAD databases who were solicited to respond using standardised text), of whom 79% were scientific researchers [5] and none were African [6]. Finally, the GSR access policy was discussed at a Genomic Variation Program Workshop on Establishing a Central Resource of Data from Genome Sequencing Projects (June 2012) [7], which also had no African representation in speakers or scheduled content, although the participant list of this workshop is not available to confirm whether Africans were present.

## Main text

It is, however, important to consider the genomic depth and breadth of African genomes and the consequent ability to genetically distinguish small populations and communities from each other, often in approximation of ethnicity or ancestral lineage [8, 9]. This inherent genomic complexity of African populations is often disregarded in Caucasian-centric policies and recommendations, and community or population-level risks may be overlooked because such re-identification of specific Caucasian communities using genomic data is unlikely. Current National boundaries in Africa were arbitrarily defined during colonisation, and multiple African populations may co-exist in a single Nation, which in some cases has resulted in tensions between different population groups.

The ability to fine-map population-level genomic data to specific communities comes with inherent community-level risks that have already been experienced by minority Indigenous populations on other continents – such as the experiences of the Havasupai Native Americans [10, 11], or the negative implications of genomic research for the San population in Southern Africa [12, 13]. History is littered with examples of opaque, invasive, and often poor quality research that has damaged vulnerable communities [14]; such as the Xavante and Yanomani populations in Brazil [15, 16], or the Indigenous populations of Australia and New Zealand [17, 18].

It is notable that three of the respondents to the NIH “Request for Information” were representing Native American Tribes, namely the Sault Ste. Marie Tribe of Chippewa Indians (submitted by Larry Jacques), the Southcentral Foundation (submitted by Denise A Dillard), and United South and Eastern Tribes Sovereignty Protection Fund (submitted by Liz Malerba). These commentaries all included strong recommendations that any genomic information should be reviewed by Tribal review boards and/or community representatives before release. Concerns were expressed that unlimited and indefinite use of genomic summary data without oversight is dangerous to the ongoing trust relationship between Tribal populations and the NIH; that ongoing and future determination of harms from genomic information collected from tribal populations must be facilitated; and that NIH program officers and scientific reviewers might push widespread data sharing in direct contradiction to tribes’ requirements as sovereign nations [6].

As with Indigenous populations in North America and Australasia, as well as other sensitive populations across the globe, full genomic summary data for identifiable African populations or communities published online without any oversight could expose these people to a high risk of discrimination or stigmatisation. As further variant-phenotype associations are discovered, allele frequencies for these variants can be assayed in different populations using GSR, and predictions made about trait prevalence in those populations. The genomic diversity and distance between different African populations is sufficiently large, even on a local scale, that genomic summary data can uniquely identify individual communities [8, 9] who can be geographically located, and associated phenotypes can be ascribed to those specific communities based on their aggregated allele frequencies. Given known examples of ethnic discrimination, violence and xenophobia within Africa [19], as well as unfortunate historical and ongoing misappropriation of genetic data to publicly denigrate African populations [20, 21], the open availability of summary genome data for distinct African ethnic groups may be unethical because of the untenable risk of harms accruing to those populations. As such, African populations should all be regarded as ‘special populations’ for the purposes of the new NIH policy to ensure they are protected from such harms, in line with conclusions drawn in that policy that privacy risks related to broad access to GSR may be heightened for some study populations. Furthermore, participants who have provided DNA samples to date are unlikely to have consented to have their data shared openly without Access Committee oversight; and specific consent for aggregate data sharing - with full participant information about potential harms - is needed from individuals as well as generally accepted representative community organisations before further sharing of these aggregated data.

Here, we propose a framework for the use of GSR from African populations that could greatly reduce the risk to African participants, whilst still facilitating the general use of African summary genomic data to inform and advance global research to identify aetiological variants and contribute to advancing health research. This framework has three components that provide options for appropriate levels of summary data use.

### Use of GA4GH beacons

In this use case, a researcher seeking to prioritise candidate disease-causing variants in another population could check whether candidate variants have been identified in African populations, if so, at what frequency, and/or whether they have been associated with a specific disease in African populations. The Global Alliance for Genomics and Health (GA4GH) Beacon protocol [22, 23] allows researchers to make limited queries as to whether a particular variant has been seen in a particular dataset, thus encouraging sharing of information without compromising privacy, with proposed extensions to include queries of variant-phenotype associations through direct online access. Query rate limits can be used to restrict abuse of the system by “walking” across the genome using thousands of queries of the same aggregated dataset, but without restricting ease of access for honest research purposes.

### Registered user access through the African genome variation database

The African Genome Variation Database (AGVD) is under development as a project of the H3Africa Informatics Network [24, 25], and aims to be a resource for exploring African variation data available to registered users. Regionally-aggregated genomic data summaries – for example for North, South, West and East Africa - can be made available for bona fide researchers who are reviewed as part the AGVD general administration for registered users. It is likely that such summaries will provide valuable allele frequency data for regional groupings without exposing communities or populations to potential harms; and that a genetic diversity metric such as Fst [26, 27] could determine an aggregation level that provides some granularity without exposing individual populations or communities.

### Access to study population pre-calculated genomic summary data through applications reviewed by an access committee

Where requests for summary data cannot be met by the processes outlined above, applications for population-specific summary data could be made through an appropriately constituted Access Committee, which should normally already be in place to administer access to individual-level genotype data where secondary data use consents are in place. It is likely that only in a small subset of cases would this detail review be required, as beacons and regional summaries should answer many of the use cases for external researchers. Should the number of requests become unmanageable for an existing access committee, a subcommittee could be constituted of individuals who are qualified to review specifically these requests under the oversight of the main committee. Where genotype data are submitted to central repositories such as the European Genome-Phenome Archive (EGA) [28], access to African genome summary data might be managed similarly to whole dataset requests in cases where Beacons or regional aggregated data do not suffice.

## Conclusions

In conclusion, genome summary data from studies of African populations can substantially enhance ongoing health research in African and rest-of-world populations, and ethical and responsible sharing of these data should be supported. Open and unregulated online exposure of genome summary data from African populations or communities, however, may expose these populations to unacceptable risks and potential harms such as those experienced by Indigenous and/or minority population groups to date. Outlined here are three levels of controlled access to genome summary data from African populations and communities that can harness the benefits of these data for global and local health research, whilst mitigating the risks and potential harms for the African participants and communities who provide their samples and data for genomic research.

## Data Availability

All information cited is openly available online and URLs are provided in the References section.

## Abbreviations

AGVD: African Genome Variation Database
EGA: European Genome-Phenome Archive
GA4GH: Genomic Alliance for Genomics and Health
GSR: Genome Summary Results
NIH: National Institutes of Health
